# Case of Tumors in the Thorax, Connected with Renal Calculi

**Published:** 1829-11

**Authors:** C. J. Roberts

**Affiliations:** Physician to the General and to the South London Dispensaries, &c.


### TUMORS IN THE THORAX.

*Case of Tumors in the Thorax, connected with Renal Calculi.* By C. J. Roberts, m.d. Physician to the General and to the South London Dispensaries, &c.

John Sadler, æt. forty-three, coal and timber porter, was admitted a patient of the South London Dispensary, on the 8th July, 1829. He was first seen, in my absence, by one of the pupils, who ordered him a blister to his left side, and some aperient medicine. Three days afterwards, my attendance was requested at his own home, he then being unable to leave his bed. I found him complaining of a very acute pain under the angle of the right scapula, which was much increased by pressure, and so severe at intervals as to cause him to cry out. The tongue was clean; the pulse soft, rather full, but not exceeding seventy-six in number; his countenance was sallow, and the conjunctiva somewhat suffused with bile. I prescribed for him calomel and opium.

In three days his mouth was sore, but the pain remained nearly the same: he had perhaps an interval of ease for two or three hours in the twenty-four, but never longer.

On the 20th of July, (four days after,) the soreness of the gums having subsided, and the pain continuing, I was induced to repeat the mercury; and, in addition, twenty leeches were applied to the part.

On the 22d July, the mouth again became somewhat sore, and the mercury was suspended, on account of his petulant complaints; but was resumed on the succeeding day, and the leeches were reapplied.

On the 25th, the mercury, notwithstanding its being combined with opium, produced diarrhœa, and it was discontinued. As he had found the greatest relief from the topical abstraction of blood, I ordered him to be cupped upon the part, and to lose sixteen ounces of blood.

On visiting him on the 28th, I found the cupping had not caused the slightest alleviation of the pain; and, at his earnest request, he had fifteen more leeches applied to the back. He continued nearly in the same state for a week, when the pain was so much aggravated, that twelve more leeches were employed, and ten grains of Pil. Sapon. cum Opio were ordered to be taken at bedtime every night.

On the 8th August, twelve more leeches were applied; and afterwards the part was rubbed with the Ung. Tart. Antim., but without relief.

9th.—Twenty-four more leeches were again ordered, and a powder of calomel, opium, and tartar emetic, to be taken at bedtime. During the whole of this period the pulse never ranged higher than ninety, and the tongue was uniformly clean.

From the interval between the 11th and the 19th of August, fifty leeches were used, at his own earnest desire, and the opium was also continued.

On the 21st August, a blister was again applied, but without effect. He was getting much thinner, and he now fancied he could feel an abscess about to point; in which opinion he was confirmed in consequence of a practitioner in the neighbourhood, who had formerly attended him, having told him that he had no chance of living unless the tumor was opened, and a vent given to the matter. On hearing this, I requested my colleague, Mr. Amesbury, to visit him, who perfectly agreed with me that it would not be safe to use the lancet, and that it would be better to apply a poultice. This plan was pursued very strictly, until he complained that the weight of the poultices inconvenienced him; when fomentations were substituted.

The same practitioner who recommended puncturing the part again saw him, and wished that the operation should be performed; but the patient had become satisfied that it would be of no service.

From this time he sank gradually, and died on the 8th of September.

About three weeks previous to his decease, he complained that his urine was small in quantity, and caused slight smarting pains on being passed. It was very high coloured, depositing a copious reddish sediment, and was loaded with mucus; but no decided nephralgic or lithic symptoms ever showed themselves.

On opening the body, the general appearance of the intestines was healthy, and, on tracing them, no marks of disease were evident. The liver, which we had supposed to be the principal seat of the mischief, was found to be healthy, except one or two small tubercles, but which were so minute as not to allow us to presume, for one moment, that these could have been the cause of the violence of the symptoms. The gall-bladder was very large, and full of very filthy black bile, to the extent of two ounces, but bore no traces of inflammation or disorganization.

The right kidney was increased to nearly or quite four times its natural size, and contained within its infundibula a quantity of stones, of different dimensions, varying from a small nut to that of a tare. They were of a black colour, and some of them were covered by a deposit of calcareous matter of a lighter colour, nearly approaching to white, and very friable. The left kidney was much diminished in size, and was studded with distinct spiculæ of bone, and patches of ossific matter, throughout its substance. There was also a stone, nearly the size of a large horsebean, impacted in one of the infundibula.

The heart was quite healthy. The lungs were entirely sound, and so perfectly did they collapse on the thorax being cut into, that they lay contracted into a very small compass upon the sides of the vertebræ. On looking at the back part of the right lung, two tumors were discovered lying on the transverse process of the dorsal vertebræ, and covered by the pleura costalis. The upper one was the smaller, and occupied the space between the fourth and seventh dorsal vertebræ; and the inferior and larger one was separated from the superior one by the space of one rib, and extended from the eighth to the eleventh dorsal vertebræ. These tumors were partly ligamento-cartilaginous, with ossific deposits, together with a very small quantity of dark-coloured pus. The bodies, and a portion of the transverse processes of the vertebræ upon which they rested, were roughened, and absorption of them was commencing. The sixth rib was dislocated, the head having been already absorbed.

The bladder was somewhat thickened, and its internal coat rather more vascular than usual, but not sufficient to have attracted attention, had that not been previously excited by the calculi in the kidney.

I was induced to believe the patient was afflicted with inflammation of the liver, which was about to terminate by suppuration, from the constant pain about the lower angle of the scapula, and from the sallow countenance and injected state of the conjunctivæ. My opinion was also strengthened from his intemperate habits being notorious, and also from his having been constantly exposed to the weather. He never complained of pain higher up in the course of the spine than about the ninth rib, and this was aggravated by firm pressure being made with the hand. Towards the termination of life, very gentle touching of the part would excite pain in it for hours. He lay constantly on his left side, or upon his face. The circumstances which most perplexed me were the very great steadiness of the pulse and the cleanness of the tongue; the former generally not varying more than from seventy-six to eighty-four until within a very short period of his decease, and then they did not exceed one hundred; and the tongue never having been under any circumstances loaded with fur.

The pain in the lumbar region was never sufficient to call our attention to it, and, when the hand was pressed on the spot, he did not complain in a manner to lead to the inference that so extensive a disease existed in its neighbourhood. In fact, the whole of the pain by which he was so much distressed resided in the tumors within the thorax. That neither opium alone nor in conjunction with calomel should mitigate his sufferings, cannot create surprise; but it was rather a curious fact that cupping, which abstracted the blood from the part in much greater quantity and with more rapidity, than the leeches, should fail in producing as great relief: perhaps, it may be presumed that the pressure of the cups caused more uneasiness than the abstraction of the blood did good. The pain must have been caused by the tumors pressing upon the nerves in the vicinity.

The tumors in the thorax were attributed by his wife to his having been kicked in that part by a man with whom he was fighting, some years since.

The calculi were analysed by my friend, Mr. Hennell, of Apothecaries' hall, who considered them to consist of phosphate of lime, and the matter deposited round them to be the ammoniaco-magnesian phosphate: they are in the collection of the Medical School in Aldersgate street.

31, *Bridge street.*

